# Estimation of salt intake from spot urine may assist the risk assessment of gastric cancer

**DOI:** 10.3164/jcbn.19-65

**Published:** 2019-11-28

**Authors:** Atsushi Goto, Jun Nishikawa, Shunsuke Ito, Eizaburou Hideura, Tomohiro Shirasawa, Koichi Hamabe, Shinichi Hashimoto, Takeshi Okamoto, Hideo Yanai, Isao Sakaida

**Affiliations:** 1 Department of Gastroenterology and Hepatology, Yamaguchi University Graduate School of Medicine, Minami-kogushi 1-1-1, Ube, Yamaguchi 755-8505, Japan; 2 Faculty of Laboratory Science, Yamaguchi University Graduate School of Medicine, Minami-kogushi 1-1-1, Ube, Yamaguchi 755-8505, Japan; 3 National Hospital Organization Kanmon Medical Center, Department of Clinical Research, Chofu-sotouracho 1-1, Shimonoseki, Yamaguchi 752-8510, Japan

**Keywords:** gastric cancer, estimated salt intake, spot urine, urine sodium, *Helicobacter pylori*

## Abstract

Daily salt intake can be estimated by measuring sodium and creatinine concentrations in spot urine. Excessive salt intake is risk factor for gastric cancer. We examined the correlation between estimated salt intake from spot urine and risk of gastric cancer. This study included gastric cancer patients who underwent treatment at our hospital and patients in whom esophagogastroduodenoscopy was performed but gastric cancer was not observed. The history of *H. pylori* infection was known in these patients. Spot urine was collected, and daily salt intake was estimated from urine sodium and urine creatinine. Mean estimated salt intake was significantly higher in 120 gastric cancer patients (9.18 g/day) than in 80 non-gastric cancer patients (8.22 g/day). Multivariate analysis revealed estimated salt intake and *H. pylori* infection to be independent risk factors for gastric cancer. Among *H. pylori*-infected patients, salt intake was significantly higher in gastric cancer patients (9.25 g/day) than in non-gastric cancer patients (8.01 g/day). In conclusion, salt intake estimated from spot urine was high in patients with gastric cancer, especially in *H. pylori* infected patients. Spot urine is a simple examination and it may be applied as a new risk assessment of gastric cancer.

## Introduction

The most established risk factor for the development of gastric cancer (GC) is *Helicobacter pylori* infection. Excessive salt intake is also a risk factor for GC development. Tsugane *et al.*^(1)^ and Shikata *et al.*^(2)^ evaluated the trend of salt intake by recording meals and prospectively examining the relationship with carcinogenesis. Excessive salt intake has been shown to be a dose-dependent risk factor for GC. Furthermore, urine sodium excretion measured by 24-h urine collection was correlated with GC mortality at the population level.^(3)^ It thus appears that GC is likely to occur in *H. pylori*-infected persons who consume highly salty foods.

Questionnaire surveys and 24-h urine collection have been performed to estimate daily salt intake, but these are laborious and burdensome for patients and examiners. The rate of failure of 24-h urine collection was reported to be 40%.^(4)^ Tanaka *et al.*^(5)^ reported that 24-h urine sodium excretion can be estimated by measuring sodium and creatinine concentrations in spot urine. This method is based on the database of Japanese people who participated in the INTERSALT Study,^(6)^ an international collaborative study on the correlation between salt intake and blood pressure. The formula is recommended as a simple and practical evaluation method in the Guidelines for the Management of Hypertension of the Japanese Society of Hypertension^(7)^ and is used as an indicator of blood pressure management in cardiovascular disease patients.^(8)^ We attempted to evaluate relationship between salt intake and GC using the estimation method of salt intake from spot urine.

## Materials and Methods

### Patients and methods

This study is cross-sectional study. The subjects comprised GC patients treated at Yamaguchi University Hospital from August 2016 to September 2018 and those with no indication of GC by esophagogastroduodenoscopy. Estimation of 24-h urine sodium excretion from spot urine in patients with stage 3–4 chronic renal failure is not accurate.^(9)^ Patients with a serum creatinine level ≥2.0 mg/dl and those taking diuretics were excluded from the study.

With the consent of the subjects, spot urine was collected once and urine sodium (mEq/L) and urine creatinine (mg/dl) were measured. Daily salt intake was estimated according to the following calculation formulae reported by Tanaka *et al.*^(5)^

$$\begin{array}{l} \text{Predicted value of 24-h urine creatinine (mg/day) }=\\ -2.04\times \text{age}+14.89\times \text{weight (kg)}+16.14\times \text{height (cm)}-2,244.45 \end{array}$$ (1)

$$\begin{array}{l} \text{Estimated 24-h urine sodium (mEq/day) }=\\ {}[21.98\times \text{Na concentration in the spot urine/creatinine concentration in the spot urine }\times \\ {\text{predicted value of 24-h urine creatinine}]}^{0.392} \end{array}$$ (2)

$$\begin{array}{l} \text{Estimated 24-h salt intake (g/day)}=\text{estimated 24-h urine sodium}\times 0.0585 \end{array}$$ (3)

We examined the correlations between estimated salt intake and presence or absence of GC, age, sex, history of alcohol consumption, history of smoking, family history of GC, and *H. pylori* infection. Patients with serum *H. pylori* IgG antibody titer of ≥10 and no history of eradication were considered currently *H. pylori*-infected patients. Patients with *H. pylori* IgG antibody titer <10 and a history of eradication were considered previously *H. pylori*-infected patients. The currently *H. pylori*-infected patients and the previously *H. pylori*-infected patients were combined into the *H. pylori* infection group. Patients with *H. pylori* IgG antibody titer <10, no history of eradication therapy, and no evidence of atrophic gastritis endoscopically were considered *H. pylori*-uninfected patients. Patients who did not fit into any category were excluded as subjects of the study because the status of *H. pylori* infection was unclear. Thus, 120 GC patients and 80 non-GC patients who fulfilled these conditions were selected as subjects. Patients with a history of alcohol consumption were those who had habitually consumed alcohol, even if they were currently abstaining from alcohol. Similarly, patients with a history of smoking were those who had habitually smoked, even if they were currently abstaining from smoking.

### Assessment of clinicopathological findings

 Clinicopathological findings of GC (i.e., macroscopic type, tumor diameter, differentiated type, depth of invasion, lymphovascular invasion, degree of atrophy, and occurrence of multiple GCs) were respectively evaluated for a correlation with estimated salt intake. Regarding macroscopic type, tumors were classified into lesions mainly comprising a protruded type and flat or depressed type. Tumor diameter was classified into lesions of ≤30 mm or lesions >30 mm. Histologically, cancers were classified into differentiated cancer and undifferentiated cancer according to the Nakamura *et al.* classification.^(10)^ When these cancers were mixed, the cancer was classified as the predominant type. Of the 120 GC cases, 118 were of early GC for which endoscopic resection was performed. Therefore, depth of invasion was classified into intramucosal invasion and submucosal or deeper invasion. According to the Kimura-Takemoto classification of atrophy, background mucosa was classified as open-type severe atrophy, closed-type mild atrophy, or non-atrophy. Multiple GC was defined when ≥2 cancers occurred synchronously or metachronously.

### Statistical analysis

In univariate analysis, Fisher’s exact test for discrete variables and *t* test for continuous variables were used. Multiple logistic regression analysis was used for multivariate analysis (Ekuseru-Toukei 2010 for Windows; Social Survey Research Information Co., Ltd., Tokyo, Japan), and each result was determined to be significantly different when *p*<0.05.

### Statement of ethics

This study was performed according to the guidelines of the Declaration of Helsinki and the study protocol was approved by the institutional Review Board of Yamaguchi University Hospital (approval number H26-119).

## Results

Patient characteristics of the 200 subjects and estimated salt intake were evaluated. The GC group comprised 120 patients, and the non-GC group comprised 80 patients. There were 148 men (74%) and 52 women (26%) whose median age was 70 (35–91) years. Median sodium concentration in spot urine was 101 (15–244) mEq/L, median creatinine concentration was 84.6 (9.6–337.9) mg/dl, and median estimated salt intake was 8.68 (2.47–17.04) g/day. History of alcohol consumption was noted in 120 patients (60%), smoking history in 125 (62.5%), and family history of GC in 42 (21%). In total, 84 (42%) were currently *H. pylori*-infected patients, 81 (40.5%) were previously infected, and 35 (17.5%) were uninfected.

Analysis results of GC patients and non-GC patients can be compared in Table 1. Mean estimated salt intake in GC patients and non-GC patients was 9.18 g/day and 8.22 g/day, respectively, and was significantly higher in GC patients. Univariate analysis revealed that patients with GC had significantly higher age and included more men and more cases of current and previous *H. pylori* infection. Multivariate analysis revealed significant differences for estimated salt intake and current or previous *H. pylori* infection.

**Fig. 1:**
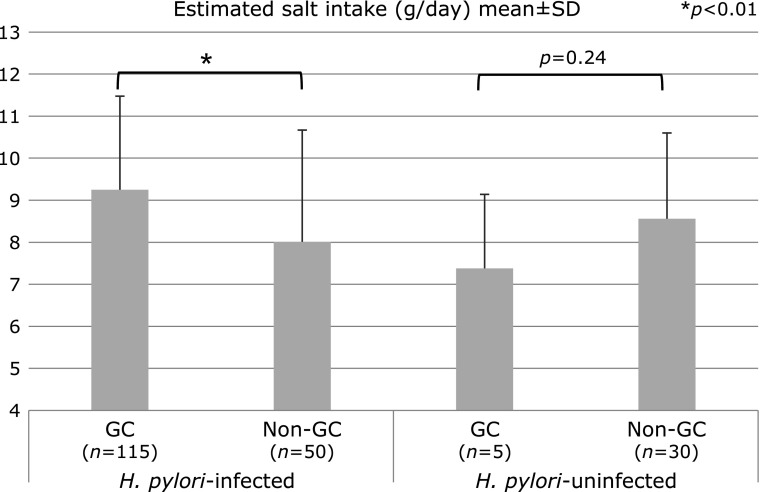
Relations between *H. pylori* infection and estimated salt intake by univariate analysis.

Figure 1 shows the correlation between estimated salt intake and *H. pylori* infection. Estimated salt intake of the *H. pylori* infection group, including currently or previously *H. pylori*-infected patients, was 9.25 g/day, which was significantly higher than the 8.01 g/day in non-GC patients. In *H. pylori*-uninfected group, estimated salt intake of GC patients was not high (7.38 g/day) compared to that in the non-GC patients (8.56 g/day). The difference was not statistically significant (*p* = 0.24).

Table 2 shows the relationship between clinicopathological features of the 120 GC lesions and estimated salt intake. No correlation between estimated salt intake and any of the factors was found.

## Discussion

We revealed that estimated salt intake and *H. pylori* infection were significantly related for GC by the multivariate analysis. This suggests that one of the GC risk may be evaluated by a simple examination such as spot urine. Tsugane *et al.*^(11)^ showed an almost linear correlation between the cumulative mortality rate of GC and the urinary salt excretion level in 24-h urine samples. Furthermore, they estimated salt intake by a questionnaire survey, and showed that high-salt diet is a dose-dependent risk of GC in middle-aged men.^(1)^ In a Netherlands cohort study, 120,852 people and 282 GC cases were investigated during the 6.3 years of follow-up by a questionnaire survey. The results suggested that dietary salt and salted meat intake were weakly positively associated with the risk of GC.^(12)^ Spot urine is an examination used in general medical checkup, thus more patients may be able to be aware of the risk of gastric cancer as well as measures against hypertension.

Two studies using spot urine were recently made to evaluate GC risk. Park *et al.*^(13)^ showed that 24-h urine sodium excretion estimated from spot urine correlates with the prevalence of GC. In the study, *H. pylori* infection was not evaluated and could not be excluded as a confounding factor. Thapa *et al.*^(14)^ showed that salt intake estimated by urinary sodium/creatinine ratio is associated with progression of GC or dysplasia in patients with persistent *H. pylori* infection at a risk ratio of 1.49. This result was originally intended for patients who were positive for *H. pylori*, and *H. pylori*-uninfected patients were not included. The novelty of our study was that the estimated salt intake was evaluated based on the presence or absence of *H. pylori* infection. We showed that estimated salt intake was significantly higher in patients with GC in the *H. pylori* infection groups, whereas we found that GC patients without *H. pylori* infection did not have high salt intake. Three patients had signet ring cell carcinomas, and two had fundic gland-type GCs. Risk factors of GCs occurring in *H. pylori*-uninfected individuals are unknown.^(15)^ Excessive salt intake might not be related to carcinogenesis in *H. pylori*-negative GC.

Kato *et al.*^(16)^ reported that salt intake induces carcinogenesis in a dose-dependent manner under *N*-methyl-*N*-nitrosourea administration in *H. pylori*-infected Mongolian gerbils. Continuous salt intake is thought to change the gastric mucus environment and promote gastric carcinogenesis in the *H. pylori* infected stomach. Reduction of salt intake combined with *H. pylori* eradication might inhibit the development of GC.

Hirata *et al.*^(17)^ reported that expression level of CD44 variant 9 (CD44v9), a functional cancer stem cell marker, was a predictor for the recurrence after the resection of early GC. Furthermore, Tsugawa *et al.*^(18,19)^ showed that accumulation of cytotoxin-associated gene A (*CagA*) in cells overexpressing Capping actin protein of muscle Z-line α subunit 1 (CAPZA1) induces the expression of CD44v9. Thus, overexpression of CAPZA1 in *H. pylori*-infected gastric mucosa is thought to increase the risk of GC. Whether high salt intake induces CAPZA1 expression is an interesting subject for future investigation.

As a limitation of the study, diurnal variation and meals on the day before urine collection may affect the value.^(20)^ When conducting patient guidance with reference to estimated salt intake from spot urine, it may be necessary to perform multiple measurements under the same urine collection conditions. Because there are few cases of *H. pylori*-uninfected GC patients, it cannot be concluded whether salt intake is not related to the carcinogenesis in *H. pylori*-uninfected patients. It is a future task to increase the number of *H. pylori*-uninfected GC patients.

In conclusion, estimated salt intake from spot urine was significantly higher in patients with *H. pylori*-positive GC. Spot urine is a very simple examination, and it may be applied as a risk assessment for GC in general medical checkup without patient burden.

## Author Contributions

AG, study concept and design, drafting of the manuscript; JN, study concept and design; SI, acquisition of data; EH, acquisition of data; TS, statistical analysis; KH, SH, TO, and HY, revision of the manuscript for important intellectual content; IS, study supervision.

## Figures and Tables

**Table 1:** Results of univariate and multivariate analyses of gastric cancer and non-gastric cancer cases

		Univariate analysis				Multivariate analysis	
		Gastric cancer (*n* = 120)	Non-gastric cancer (*n* = 80)	*p* value		Odds ratio (95% confidence interval)	*p* value
Age	Years (mean)	70.9	66.3	0.003		1.03 (0.99 to 1.06)	0.13
Sex							
	Male	95	53	0.049		2.39 (0.94 to 6.09)	0.067
	Female	25	27				
Drinking history							
	Presence	74	46	0.56		0.79 (0.35 to 1.81)	0.58
	Absence	46	34				
Smoking history							
	Presence	75	50	1		0.8 (0.34 to 1.91)	0.62
	Absence	45	30				
Family history of gastric cancer							
	Presence	30	12	0.11		1.08 (0.46 to 2.53)	0.86
	Absence	90	68				
*H. pylori* infection							
	Current infection	51	33	2.3 × 10^−10^		7.94 (2.63 to 23.9)	2.4 × 10^−4^
	Previous infection	64	17			17.7 (5.69 to 55.2)	7.2 × 10^−7^
	Uninfected	5	30				
Estimated salt intake (g/day) (mean)		9.18	8.22	0.005		1.16 (1.01 to 1.35)	0.048

**Table 2:** Relations between clinicopathologic factors of gastric cancer and estimated salt intake by univariate analysis

		(*n* = 120)	Estimated salt intake (g/day) (mean)	*p* value
Macroscopic type				
	Protruded	38	9.01	0.59
	Depressed	82	9.25	
Tumor diameter (mm)				
	≤30	105	9.27	0.23
	>30	15	8.52	
Differentiated type				
	Differentiated	112	9.24	0.23
	Undifferentiated	8	8.25	
Depth of invasion				
	Intramucosal	97	9.1	0.46
	Submucosal or deeper	23	9.49	
Lymphovascular invasion				
	Absence	111	9.14	0.57
	Presence	9	9.59	
Degree of atrophy				
	Closed type or non-atrophy	28	8.83	0.35
	Open type	92	9.28	
Multiple gastric cancer				
	Single gastric cancer	80	8.91	0.07
	Multiple gastric cancer	40	9.7	
